# Updated Immunotherapy for Gastric Cancer

**DOI:** 10.3390/jcm12072636

**Published:** 2023-04-01

**Authors:** Yukiya Narita, Kei Muro

**Affiliations:** Department of Clinical Oncology, Aichi Cancer Center Hospital, Nagoya 464-8681, Japan

**Keywords:** chemotherapy, gastric cancer, immune checkpoint inhibitor, programmed cell death 1

## Abstract

Gastric cancer treatments are evolving rapidly. For example, immune checkpoint inhibitors, especially those that target PD-1 or PD-L1, have long-term efficacy in a subset of gastric cancer patients, and are currently the first-line therapy. Immunotherapies approved for use in untreated gastric cancer patients include monotherapy and chemotherapy-immunotherapy combinations. Major clinical trials have reported efficacy and safety data suggesting that PD-L1 expression is important for regimen selection, although other biomarkers, clinicopathologic factors, and patient preference might also be relevant in other situations. Currently, several novel biomarkers and therapeutic strategies are being assessed, which might refine the current treatment paradigm. In this review, we describe the current treatment regimens for patients with gastric cancer and detail the approach we use for the selection of first-line immunotherapy regimens.

## 1. Introduction

Gastric cancer is the fourth most common cancer and the fourth leading cause of cancer death globally [1]. The standard of care for advanced gastric cancer is first-line platinum doublet treatment. However, the prognosis remains poor at 8–15 months after initial treatment [2,3,4,5,6,7,8]. The morbidity and mortality rates for stomach cancer are high in East Asian countries, including Japan and Korea, Eastern Europe, and South America. There are differences in the frequency of esophagogastric junction cancer, five-year survival rates, perioperative adjuvant therapy, and standard chemotherapeutic regimens between countries in Asia and the West [9]. Of note, the endoscopic diagnosis of early gastric cancer is high in Japan, and endoscopic mucosal resection/dissection is widely used. Regarding surgery, the degree of lymph node dissection tends to be lower in Europe and the USA compared with Asian countries. Furthermore, the frequency of esophagogastric junction cancer is high in Europe and the USA. Indeed, clinical trials report about 25–30% or more of all cases are esophagogastric junction cancer (Table 1).

**Figure 1:**
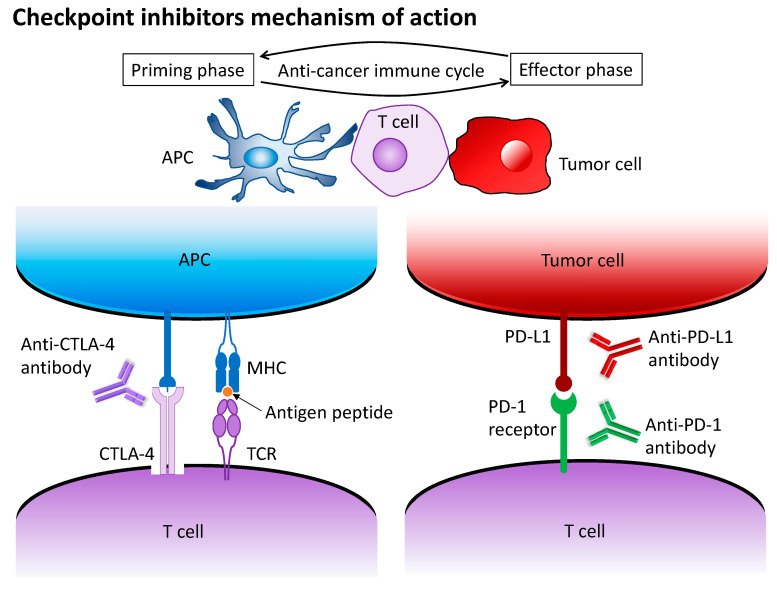
Mechanisms of immune checkpoint inhibitors, such as CTLA-4, PD-1, and PD-L1.

The development of immune checkpoint inhibitors has caused a paradigm shift regarding the treatment of many cancers. Blocking the programmed cell death (PD)-1 protein, its ligand (PD-L1), and cytotoxic T-lymphocyte-associated protein 4 (CTLA-4) was reported to achieve durable responses in advanced gastric cancer patients (Figure 1) [10,11,12,13,14,15]. Several PD-(L)1 inhibitors for advanced gastric cancer, including pembrolizumab, nivolumab, and sintilimab, have been approved in first-line or third-line settings in Europe, the USA, and Asia (Table 2). Here, we review the current status of immunotherapy for advanced gastric cancer and discuss potential future directions.

Antigen presentation by MHC class II molecules on APCs is required for T-cell activation. A second activation signal is required, which can be blocked by CTLA-4 binding to CD80 or CD86. Anti-CTLA-4 antibodies, such as ipilimumab, block the negative regulatory signal for CTLA-4 binding. Another immune checkpoint is the PD-1 receptor expressed on activated T cells, which binds to the PD-L1 ligand expressed on tumor cells, which promotes T cell apoptosis. Anti-PD-1 and anti-PD-L1 antibodies, such as nivolumab, pembrolizumab, and sintilimab, inhibit this signal.

Abbreviation: PD-1, programmed death-ligand 1; PD-L1, programmed cell death protein 1; CTLA-4, cytotoxic T-lymphocyte-associated protein 4; APC, antigen presenting cell; TCR, T cell receptor; MHC, major histocompatibility complex.

## 2. Genetic and Molecular Profiles and Immunotherapy in Gastric Cancer

Biomarkers for immune checkpoint inhibitors can be categorized into three major groups: Immunological, genetic, and virological [16]. The expression of PD-L1 before treatment can be used as an immunological biomarker that is predictive of tumor shrinkage. The tumor mutation burden, which reflects the neoantigenic diversity of a tumor, might also be used to predict the benefit of immunotherapy related to the high anti-PD1 response rate in DNA mismatch repair deficient (dMMR) tumors. Virus-associated cancers express highly immunogenic viral neoantigens that are involved in therapeutic responses to immunotherapy. These three types of biomarkers are closely associated with the molecular pathology classification of gastric cancer. The Cancer Genome Atlas Research Network (TCGA) has proposed genetic and molecular classifications for gastric cancer by the use of four subtypes: Tumors positive for Epstein–Barr virus (EBV), tumors with microsatellite instability (MSI-H), tumors with chromosomal instability (CIN), and genomically stable tumors (GS) [17]. EBV-positive gastric cancer is characterized by the extensive infiltration of CD8-positive cytotoxic T cells and high numbers of dendritic cells. In the TCGA, the *CD274* and *PDCD1LG2* genes, which encode PD-L1 and PD-L2, respectively, were frequently amplified in EBV-positive type. The TCGA and Asian Cancer Research Group (ACRG) indicated that the MSI-H or dMMR gastric cancer subtypes have a high frequency of mutations and hypermethylation, which contribute to the increased expression of neoantigens. The MSI-H subtype is also characterized by the high numbers of CD8+ T cells infiltrating into the tumor microenvironment, leading to the high expression of PD-L1 on tumor cells and tumor-infiltrating lymphocytes. After the resection of gastric cancer, MSI-H cases were reported to have a markedly better prognosis than MSS cases [18,19,20,21,22,23]. An integrated analysis of phase III trials (including the MAGIC and CLASSIC trials) investigating the perioperative adjuvant therapy of MSI-H patients reported that adjuvant therapy, including fluoropyrimidines did not have an additive effect on disease-free survival (DFS) or overall survival (OS) compared with surgery alone [24]. The National Comprehensive Cancer Network guidelines recommend universal screening for MSI status [25]. A transcriptome analysis by the TCGA showed that immune cell signaling was significantly upregulated in EBV-positive or MSI-H subtypes compared with the GS or CIN subtypes.

Numerous recent clinical trials of immune checkpoint inhibitors for the treatment of gastric cancer reported that the combined positive score (CPS), defined as the number of cells stained positive for PD-L1 (tumor cells, lymphocytes, macrophages) as a proportion of the total number of tumor cells multiplied by 100, is useful for predicting treatment responses [10,13,14,26,27,28,29,30]. The PD-L1 CPS score is commonly used as a stratification marker in clinical trials, and MSI-H and EBV-positive gastric cancers were demonstrated to have high PD-L1 CPS scores [31]. 

## 3. Anti-PD-1/PD-L1 Antibodies Plus Chemotherapy as First-Line Therapy

**Figure 2:**
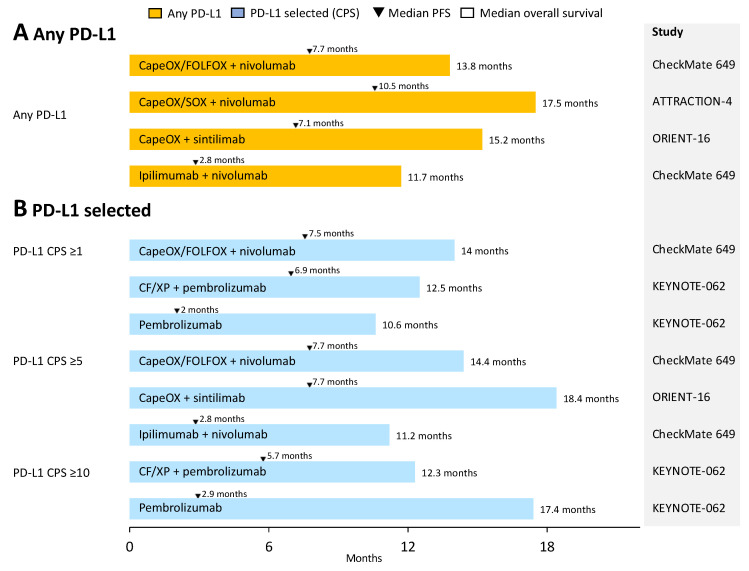
Upfront immunotherapy of patients with advanced gastric cancer. The left side shows PD-L1 status. The middle bar indicates the median OS, and the black triangle indicates the median PFS. On the right side, the clinical trials are listed. (**A**) Immune checkpoint inhibitors plus cytotoxic drugs or dual blockade with ipilimumab and nivolumab, irrespective of PD-L1 expression. (**B**) Immune checkpoint inhibitors plus cytotoxic drugs, single-agent immune checkpoint inhibitors, or dual blockade with ipilimumab and nivolumab on the basis of PD-L1 CPS. Abbreviation: PD-L1, programmed cell death protein 1; CapeOX, capecitabine + oxaliplatin; XP, capecitabine + cisplatin; FOLFOX, fluoropyrimidine + leucovorin + oxaliplatin; SOX, S-1 + oxaliplatin; PFS, progression-free survival; CF, cisplatin + fluoropyrimidine.

PD-1 and PD-L1, cell surface proteins involved in suppression of the immune system, are termed immune checkpoints and limit a host’s ability to kill tumors. Anti-PD-1/PD-L1 inhibitors, termed checkpoint inhibitors, block PD-1 and PD-L1 interactions and activity. Figure 2 summarizes clinical trials that have investigated the use of immune checkpoint inhibitors as first-line chemotherapy for patients with advanced gastric cancer. Four pivotal phase III trials (KEYNOTE-062, CheckMate 649, ATTRACTION-4, ORIENT-16) to evaluate the efficacy of immune checkpoint inhibitors in primary chemotherapy for unresectable advanced gastric cancer have been published. These trials differed in terms of their inclusion criteria, combination of cytotoxic drugs, trial design, statistical settings, and geographic regions.

The KEYNOTE-062 trial was the first study to evaluate the benefit of immune checkpoint inhibitors by evaluating the non-inferiority of pembrolizumab to chemotherapy (XP, capecitabine plus cisplatin; or CF, cisplatin plus fluoropyrimidine) and the superiority of pembrolizumab plus chemotherapy in patients with HER2-negative, PD-L1-positive (CPS ≥ 1) advanced gastric or esophagogastric junction cancer [13,28]. The co-primary endpoints were OS in the CPS ≥ 1 or CPS ≥ 10 patient populations and progression-free survival (PFS) in the CPS ≥ 1 patient population, via the analysis of measurable lesions. No advantage in PFS (median PFS, 6.9 vs. 6.4 months; hazard ratio [HR], 0.84 [95% confidence interval (CI), 0.70–1.00]; *p* = 0.04) or OS (median OS, 12.5 vs. 11.1 months; HR, 0.85 [95% CI, 0.70–1.03], *p* = 0.05) was noted for the pembrolizumab plus chemotherapy arm over the chemotherapy arm in a CPS ≥ 1 patient population. The chemotherapy plus pembrolizumab also showed no advantage in OS and PFS in the CPS ≥ 10 patient population.

The CheckMate 649 global phase III trial evaluated the superiority of nivolumab plus ipilimumab or nivolumab plus chemotherapy over chemotherapy (CapeOX, capecitabine + oxaliplatin; or FOLFOX, fluoropyrimidine + leucovorin + oxaliplatin) in patients with HER2-negative gastric, esophagogastric junction, or esophageal adenocarcinoma [10,26]. The PFS and OS in the CPS ≥ 5 patient population were set as the co-primary endpoints. Twenty-four percent of enrolled patients were Asian and 60% had PD-L1 CPS ≥ 5 tumors. The primary endpoint of PFS (median PFS, 7.7 months vs. 6.0 months; HR, 0.68 [95% CI, 0.56–0.81]; *p* < 0.0001) and OS (median OS, 14.4 months vs. 11.1 months; HR, 0.71; 95% CI, 0.59–0.86; *p* < 0.0001) in the CPS ≥ 5 patient population was significantly prolonged in the nivolumab plus chemotherapy arm compared with the chemotherapy arm. A significant prolongation in the OS was also demonstrated in the overall randomized population (13.8 vs. 11.6 months; HR, 0.80 (95% CI, 0.68–0.94); *p* = 0.0002). Based on these results, nivolumab plus chemotherapy was approved in the USA, Japan, Korea, China, and Taiwan for patients with advanced gastric cancer regardless of their PD-L1 CPS, and in Europe for patients with CPS ≥ 5.

The ATTRACTION-4 trial was a randomized placebo-controlled phase III study conducted in Asian countries (Japan, Korea, and Taiwan) [32] to determine any benefit of adding nivolumab to chemotherapy (SOX, S-1 + oxaliplatin; or CapeOX) in patients with HER2-negative advanced gastric or esophagogastric junction cancer. This study and the KEYNOTE-062 trial required target lesions. The co-primary endpoints were PFS and OS, which showed prolonged PFS (median PFS, 10.5 months vs. 8.4 months; HR, 0.85 [95% CI, 0.51–0.90]; *p* = 0.0007) but not OS (median OS, 17.5 months vs. 17.1 months; HR, 0.85 [95% CI, 0.75–1.08]; *p* = 0.257). Of note, the OS of the control group in this trial was unusually high.

The ORIENT-16 trial was a randomized, placebo-controlled phase III clinical trial conducted in China that consisted of 650 patients (PD-L1 CPS ≥ 5, 61%) with advanced gastric adenocarcinoma [33]. Chemotherapy (CapeOX) plus sintilimab (PD-1 inhibitor) was superior to chemotherapy plus placebo in the CPS ≥ 5 population (median OS, 18.4 months vs. 12.9 months; HR 0.66 [95% CI, 0.51–0.86]; *p =* 0.0023) and OS in all randomized populations (median OS, 15.2 months vs. 12.3 months; HR, 0.77 [95% CI, 0.63–0.94]; *p* = 0.0090), prolonged PFS, and improved overall response rate (ORR).

Unfortunately, conflicting results have been reported from these four large clinical trials. The HR for PFS was better in the ATTRACTION4 trial than that in the CheckMate 649 (all patients) and KEYNOTE-062 trials (CPS ≥ 1). In addition, the ORR was consistently approximately 10% higher with concomitant immune checkpoint inhibitors compared with chemotherapy alone. According to these results, the short-term drug efficacy measured by the PFS and ORR was consistent across all trials. However, only the CheckMate 649 and ORIENT-16 trials demonstrated a significant improvement in the OS when adding immune checkpoint inhibitors. However, several factors should be considered when comparing these results.

Only the KEYNOTE-062 study investigated cisplatin as a combination platinum agent. The preclinical study reported oxaliplatin-induced immunogenic cell death by releasing tumor antigens, which led to the secretion of the danger-related molecules, high mobility group box 1, and adenosine triphosphate [34,35]. Cisplatin can modulate the activity of different immune cell subsets and immune phenotypes of tumor cells by enhancing antigen presentation and downregulating PD-L1 expression [34]. In head and neck or esophageal squamous cell carcinoma, immune checkpoint inhibitors in combination with fluoropyrimidine and cisplatin therapy were reported to contribute to a prolongation of survival [36,37]. Differences in backbone chemotherapy between cisplatin and oxaliplatin were not sufficient to explain the impact on the outcomes of combination treatment with chemotherapy and immune checkpoint inhibitors in trials of advanced gastric cancer.

The KEYNOTE-062 and CheckMate 649 trials were global and included 24% Asian patients. In contrast, the ATTRACTION-4 and ORIENT-16 trials were performed only in Asian countries. When considering clinical trials of gastric cancer patients, differences in the environment surrounding gastric cancer related to geographic regions must be taken into account. In Asia, gastric carcinoma is often caused by chronic gastritis due to *Helicobacter pylori*, and the diffuse type is the most common histological type. However, in Europe and North America, *Helicobacter pylori* infection rates are low, and the preferred site of gastric carcinoma is the cardia. Thus, the intestinal type is more common. Indeed, the frequency of the histological diffuse type was more common in the ATTRACTION-4 trial (51%) than in the KEYNOTE-062 (40%) and CheckMate 649 trials (29%). In contrast, numbers of patients with primary gastroesophageal junction carcinoma were lower in Asian trials (9–19%) compared with global trials (18–31%). Nevertheless, 12% of cases in the CheckMate 649 trial had esophageal adenocarcinoma, and 26% of patients in the ATTRACTION-4 trial had an unknown primary tumor. In the AVAGAST trial conducted more than a decade ago, the frequency of patients who received chemotherapy after disease progression varied widely, similar to these four recent clinical trials (64–68% in the Asian trial vs. 33–54% in the global trial) [38]. Moreover, 14% of patients in the KEYNOTE-062 trial and 9% of patients in the Checkmate 649 trial received immunotherapy in the control arm, whereas 27% of patients in the ATTRACTION-4 trial received immunotherapy [39]. These differences in primary sites, multiple lines of treatment, and tumor volume at diagnosis, as well as the widespread use of gastrointestinal endoscopy in Asian counties, may contribute to the longer prognosis of Asian patients. The survival rate of the control arm, especially in the ATTRACTION-4 trial, was excellent (17.2 months). 

Regarding genetic factors, somatic gene mutations and amplifications in key oncogenes including *KRAS*, *ERBB2*, *EGFR*, and *FGFR2* are similar in Asians and non-Asians [40,41,42]. However, differences in tumor immune characteristics have been noted. Western gastric cancers generally have high levels of T cell markers (CD3, CD45R0, CD8) and low numbers of regulatory T cells, and low levels of inflammatory cell markers (CD66b, CD68) compared to Asian gastric cancers [43]. From an immunity standpoint, the prognosis may be worse for non-Asians than for Asians. However, no prospective clinical studies have established a definitive biomarker for the addition of immune checkpoint inhibitors to chemotherapy.

**Figure 3:**
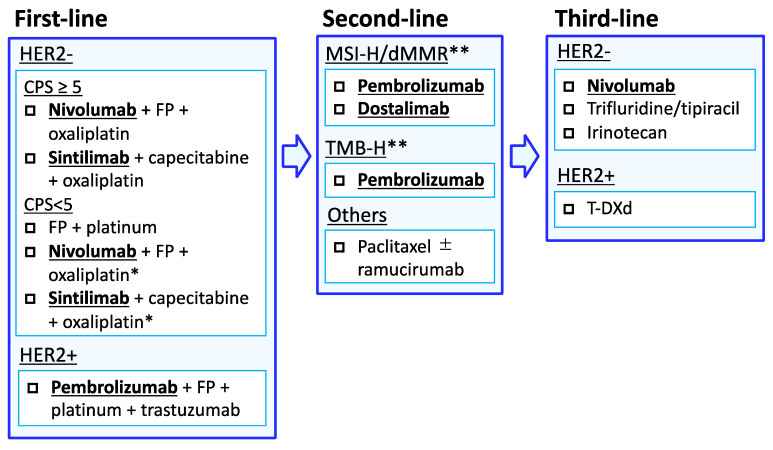
Treatment algorithm for advanced gastric cancer. The management of patients with advanced gastric cancer is primarily based on PD-L1 CPS and HER2 status. * Combination of nivolumab or sintilimab with chemotherapy may depend on a patient’s general condition, personal or institutional experience, approval status, adverse events, cost-effectiveness, and accessibility to subsequent immunotherapy for chemotherapy-naïve advanced gastric cancer patients with CPS < 5 tumors. ** Paclitaxel plus ramucirumab also is a standard regimen for MSI-H or TMB-H gastric cancer. Abbreviation: FP, fluoropyrimidine; MSI-H, microsatellite instability high; dMMR, mismatch repair deficient; HER2, human epidermal growth factor receptor 2; CPS, combined positivity score; T-DXd, trastuzumab deruxtecan.

Treatment algorithms for advanced gastric cancer in first-line settings are summarized in Figure 3. A combination of an immune checkpoint inhibitor, nivolumab or sintilimab, with oxaliplatin-based chemotherapy has been established as the standard of care for Asian and non-Asian advanced gastric cancer patients with CPS ≥ 5. However, no studies have compared a combination of sintilimab with chemotherapy alone for CPS < 5, and survival benefit of adding nivolumab was not observed in the ATTRACTION-4 trial. Based on these results, clinicians should decide whether to select immune checkpoint inhibitors in combination with chemotherapy, taking into account the patient’s general condition and subsequent treatment, as discussed later in this article.

## 4. Anti-PD-1/PD-L1 Antibody Monotherapy

### 4.1. First-Line Therapy

In the KEYNOTE-062 trial, the OS in the pembrolizumab arm was non-inferior to chemotherapy in patients with CPS ≥ 1 (median OS, 10.6 months vs. 11.1 months; HR, 0.90 [95% CI, 0.75–1.08]) with 54 months of follow-up [13,44]. Additionally, pembrolizumab monotherapy had clinically meaningful OS benefits in the CPS ≥ 10 population (median OS, 17.4 months vs. 10.8 months; HR, 0.62 [95% CI, 0.45–0.86]). Furthermore, for patients with CPS ≥ 10, the 24-month OS rate was greater in the pembrolizumab arm (28.3%) versus the chemotherapy arm (21.2%). Kaplan–Meier curves for OS crossed in the first few months, suggesting earlier death and long-term survival with pembrolizumab treatment, and the curves were maintained in a slightly crossed form in a recent update analysis.

A phase III clinical trial (JAVELIN Gastric 100) of avelumab, a PD-L1 inhibitor, compared avelumab maintenance therapy with continued chemotherapy or best supportive care after induction therapy [45,46]. The primary endpoint of OS superiority of avelumab maintenance over chemotherapy (median OS, 10.4 months vs. 10.9 months; HR, 0.91; *p* = 0.1779) was not demonstrated, nor was a prolonged duration of response. However, an exploratory subgroup analysis of CPS ≥ 1 with 22C3 antibodies showed a trend toward better use of avelumab (median OS, 14.9 months vs. 11.6 months; HR, 0.72). 

In summary, the choice of immune checkpoint inhibitor monotherapy as primary treatment for advanced gastric cancer based solely on CPS ≥ 10 may increase the risk of early progression or early death.

### 4.2. Second or Later-Line Therapy

The KEYNOTE-061 trial did not report a prolongation of the PFS (median PFS, 1.5 months vs. 4.1 months; HR, 1.27) or OS (median OS, 9.1 months vs. 8.3 months; HR, 0.82; *p* = 0.0421) for advanced gastric cancer patients with CPS ≥ 1 in the pembrolizumab arm compared with the paclitaxel arm in a second-line setting [15]. Similar to the KEYNOTE-062 trial, Kaplan–Meier curves for the OS were crossed, indicating heterogeneity in its prognostic ability.

The JAVELIN Gastric 300 trial investigated avelumab versus the physician’s choice of chemotherapy (irinotecan or paclitaxel) as third-line therapy. However, it failed to show survival benefit in the avelumab arm (median OS, 5.0 months vs. 4.6 months; HR, 1.10; *p* = 0.81) [47].

In the Asian phase III ATTRACTION-2 trial, nivolumab therapy showed survival benefit compared with placebo for patients with heavily-pretreated advanced gastric cancer, with an ORR of 11.4% (median OS, 5.3 months vs. 4.1 months; HR, 0.63 [95% CI, 0.51–0.78], *p* < 0.001) [11]. Based on these results, nivolumab monotherapy was approved in Asian countries.

In the KEYNOTE-059 study, pembrolizumab had an ORR of 15.5% for CPS > 1 and 6.4% for CPS ≤ 1 as third or later-line treatment [14]. However, the Food and Drug Administration (FDA) withdrew the indication of pembrolizumab monotherapy in 2021 after post-marketing surveillance failed to demonstrate any benefit.

Recently, a combination of immune checkpoint inhibitors with chemotherapy was approved globally for first-line treatment. However, the significance of rechallenge with nivolumab alone as third or later-line therapy for advanced gastric cancer patients previously treated with immune checkpoint inhibitors is unclear.

## 5. Anti-PD-1/PD-L1 Antibodies Plus Anti-CTLA-4 Antibodies

CTLA-4 is an immune checkpoint receptor that negatively regulates immune responses. The inhibition of CTLA-4 directly releases T cell suppression by regulatory T cells, resulting in long-term antitumor effects [48,49]. A combination of anti-CTLA-4 and anti-PD-1/PD-L1 antibodies promoted an impressive, durable response for various types of tumors [50,51,52,53]. 

In the CheckMate 032 study, a combination of ipilimumab and nivolumab for previously-treated advanced gastric cancer had a higher ORR (24% vs. 8%) in the nivolumab 1 mg/kg plus ipilimumab 3 mg/kg cohort than in the nivolumab 3 mg/kg plus ipilimumab 1 mg/kg cohort [12]. However, in the first-line CheckMate 649 trial, the ipilimumab plus nivolumab arm did not achieve the primary endpoint of OS prolongation compared with the chemotherapy arm in the CPS ≥ 5 cohort (median OS, 11.2 months vs. 11.6 months; HR, 0.89) [10,26]. Nivolumab plus ipilimumab did not provide PFS benefit (median PFS, 2.8 months vs. 6.3 months) or an increased ORR (27% vs. 47%) in patients with CPS ≥ 5. The OS was not tested statistically, and the median OS was similar between the two arms (median OS, 11.7 months vs. 11.8 months; HR, 0.91; 1-year OS rates, 49% vs. 49%). However, similar to the phenomenon reported in the immunotherapy arm of the KEYNOTE-062 and KEYNOTE-061 trials, the combination of nivolumab and ipilimumab increased the early mortality rate compared with chemotherapy, and the Kaplan–Meier curves crossed at 12 months OS, indicating a better trend in the nivolumab plus ipilimumab group after 12 months. Moreover, the median duration of response in the nivolumab plus ipilimumab arm outperformed that of the chemotherapy arm in CPS ≥ 5 (median duration of response, 13.2 months vs. 6.9 months) and all randomized populations (median duration of response, 13.8 months vs. 6.8 months). 

The randomized phase II MOONLIGHT trial investigated the efficacy of FOLFOX plus nivolumab 240 mg every 2 weeks and ipilimumab 1 mg/kg every 6 weeks compared with FOLFOX followed by nivolumab ipilimumab [54,55]. The OS of FOLFOX plus nivolumab and ipilimumab administered in parallel was markedly longer than that of FOLFOX followed by nivolumab and ipilimumab (16.46 months vs. 6.87 months) [56].

In the randomized phase II DURIGAST trial, anti-PD-L1 antibody durvalumab plus anti-CTLA-4 antibody tremelimumab with FOLFIRI did not meet the primary endpoint PFS rate at 4 months compared with durvalumab plus FOLFIRI (PFS rate at 4 months, 57.8% vs. 44.7%) [57]. However, disease control over 1 year was markedly improved in the durvalumab plus tremelimumab arm (15.2% vs. 4.3%) [58].

On the basis of these clinical trial results, a combination of PD-1/PD-L1 and CTLA-4 blockade was not indicated for all cases with advanced gastric cancer. However, immunotherapy combinations may be narrowly adapted to specific populations (see below).

## 6. Anti-PD-1/PD-L1 Antibodies Plus Anti-HER2 Therapy

Preclinical and clinical studies have demonstrated a synergistic effect when immune checkpoint inhibitors are combined with anti-Human Epidermal Growth Factor Receptor 2 (HER2) therapy to treat HER2-positive breast or gastric cancers [59,60,61]. The KEYNOTE-811 trial evaluated the efficacy of pembrolizumab in combination with anti-HER2 therapy (trastuzumab) as a first-line chemotherapy strategy for patients with HER2-positive advanced gastric cancer compared with placebo [62,63]. In the protocol-specified first interim analysis, the addition of pembrolizumab to trastuzumab and chemotherapy achieved a marked tumor reduction compared with trastuzumab and chemotherapy (ORR, 74.4% vs. 51.9% by an independent central review, *p* = 0.00006). The complete response rates were also markedly higher after adding pembrolizumab (11.3% vs. 3.1%). Furthermore, the tumor shrinkage was greater in the pembrolizumab arm than in the placebo arm (median change of tumor diameter from baseline, 65% vs. 49%). The combination of pembrolizumab to trastuzumab and chemotherapy was approved for HER2-positive gastric cancer in the USA.

Margetuximab is an anti-HER2 antibody containing an optimized Fc domain that activates innate and adaptive immune systems by an antibody-dependent cellular cytotoxicity mechanism and anti-HER2-targeted T-cell responses. Margetuximab and pembrolizumab had clinical benefits with an ORR of 18.48% in a single-arm phase Ib/II trial [64]. The phase II/III MAHOGANY trial investigated a combination of margetuximab with the anti-PD-1 antibody retifanlimab and anti-PD-1/anti-LAG3 bispecific antibody tebotelimab with or without chemotherapy. In the margetuximab plus retifanlimab cohort, a fairly good antitumor effect was observed (ORR, 64.8%) [65]. 

Trastuzumab deruxtecan (T-DXd) is an antibody-drug conjugate composed of an anti-HER2 monoclonal antibody, a cleavable tetrapeptide-based linker, and a topoisomerase I inhibitor payload [66]. T-DXd increased the number of tumor-infiltrating CD8+ T cells and enhanced PD-L1 expression and MHC class I expression on tumor cells [67]. The DESTINY-Gastric03 trial was a multicohort phase Ib/II trial for advanced HER2-positive gastric cancer [68]. In part 1, a combination of T-DXd plus fluoropyrimidine demonstrated encouraging tumor responses (50.0%) with manageable toxicity. Part 2 to evaluate T-DXd with chemotherapy +/− durvalumab is currently ongoing.

The INTEGA trial investigated the effect of trastuzumab and nivolumab in combination with FOLFOX or ipilimumab for HER2-positive esophagogastric cancer [69]. The 12-month OS rate of the primary endpoint was greater in the trastuzumab, nivolumab, and FOLFOX arm than in the trastuzumab, nivolumab, and ipilimumab arm (OS rate at 12-months, 70% vs. 57%) with a higher incidence of autoimmune-related adverse events in the ipilimumab arm (≤2% vs. ≤10%).

In summary, the combination of pembrolizumab with trastuzumab plus chemotherapy has promising efficacy in HER2-positive advanced gastric cancer in upfront settings. The OS update from the KEYNOTE-811 trial is awaited.

## 7. Perioperative/Curative Immunotherapy in Locally Advanced Gastric Cancer

The randomized phase II DANTE trial evaluated atezolizumab with FLOT chemotherapy for the treatment of resectable gastric cancer [70]. The addition of atezolizumab to FLOT as a perioperative therapy demonstrated a high pathological response (TRG1a/b) with higher PD-L1 CPS expression (atezolizumab plus FLOT vs. FLOT alone [all/CPS ≥ 5], 24%/30% vs. 15%/24%) [71]. 

Currently, critical randomized phase III trials to evaluate PD-1/PD-L1 inhibitors for locally advanced gastric cancer in perioperative or adjuvant settings are ongoing. In the KEYNOTE-585 (NCT03221426) and MATTERHORN (NCT04592913) trials, the addition of PD-1/PD-L1 inhibitors to chemotherapy is being evaluated in a perioperative setting, and the ATTRACTION-5 (NCT03006705) trial is investigating an adjuvant therapy with nivolumab plus S-1/CapeOX for Stage III gastric cancer patients.

## 8. Toxicity Profile of Immune Checkpoint Inhibitors

Immune checkpoint inhibitors are generally well tolerated. However, serious and sometimes life-threatening treatment-related adverse events (TRAEs) have been reported, with treatment-related deaths occurring in 2% of patients. The majority of TRAEs result from a non-specific reaction associated with autoimmunity during the induction of autologous tissue destruction [72]. Approximately 5–10% of grade 3 or 4 TRAEs were caused by anti-PD-1/PD-L1 antibodies in clinical trials of advanced gastric cancer [11,14]. A pivotal phase III study with a first-line setting of advanced gastric cancer, grade 3 or 4 TRAEs were increased up to about 10% in the immunochemotherapy arm compared with chemotherapy alone [10,13,26,27,32]. Although interstitial pneumonia and myocardial damage are of concern for treatment with anti-PD-1 antibodies combined with anti-HER2 therapy, no new adverse events were identified in the KEYNOTE-811 trial [63]. However, grade 3 or 4 TRAEs (approximately 30–40% of cases) were more common after treatment with anti-PD-1/PD-L1 antibodies plus anti-CTLA-4 antibodies compared with anti-PD-1/PD-L1 antibody monotherapy [26,52]. Even so, most TRAEs are manageable with systemic corticosteroids and other ancillary medications.

## 9. Molecular and Genetic Biomarkers in Gastric Cancer

### 9.1. PD-L1 CPS

The PD-L1 CPS score was reported to be a predictive marker of the efficacy of immune checkpoint inhibitors for advanced gastric cancer, and it has also been used as a stratification factor in clinical trials. However, several studies of different solid tumors reported the PD-L1 expression of tumor proportion score (TPS) but not that of CPS. The proportion of CPS ≥ 5 in general gastric cancer patients in clinics was reported to be approximately 30–40% [73,74,75]. However, CPS cutoff values that predict the effect of immune checkpoint inhibitors have not been established. According to the data from the KEYNOTE-062 trial, a CPS ≥ 10 was useful in the pembrolizumab arm [13]. On the other hand, a cutoff value of CPS 5 is recommended when adding nivolumab to chemotherapy as a first-line treatment according to the National Comprehensive Cancer Network guideline, European Society for Medical Oncology guideline, and Japanese gastric cancer guideline. [25,76,77]. 

However, there are some limitations when using CPS evaluation for gastric cancer. The 22C3 assay is used to evaluate predictors of pembrolizumab. In contrast, the 28-8 PD-L1 antibody was used in the nivolumab trials. The KEYNOTE-061 trial used the 22C3 assay, and 30% of tumors had a CPS ≥ 5. In the CheckMate 649 trial, more than 60% of cases had a CPS ≥ 5, as assessed by the 28-8 assay [78]. These different proportions reported by the KEYNOTE-061 and CheckMate 649 trials might be explained by the fact that patients with any CPS value were enrolled in the KEYNOTE-061 trial. In contrast, in the CheckMate 649 trial, all patients were enrolled at the start of the trial, and then CPS ≥ 5 was used. The independent development of PD-L1 assays in clinical trials makes it difficult to determine whether the interchangeability of the 22C3 and 28–8 assays is useful in clinical settings. Moreover, several cohort studies of gastric cancer reported excellent concordance rates between the 22C3 and 28–8 assays, although these were not perfect [73,75]. CPS is generally used to evaluate biopsy tissue from the primary tumor, but biopsy assessment may not reflect the overall tumor status because of the tumor heterogeneity [79]. Thus, the CPS is a useful biomarker for advanced gastric cancer patients treated with immune checkpoint inhibitors in a first-line setting, although it is not perfect. 

### 9.2. MSI-H

dMMR/MSI-H cancers of the colon are associated with favorable survival rates and may predict the ineffectiveness of fluorouracil-based adjuvant chemotherapy [80]. Moreover, for gastric cancer, the MSI status was reported as a favorable prognostic factor for curative disease, a negative predictor of responses to perioperative cytotoxic chemotherapy such as fluoropyrimidines, and a positive predictor of responses to immune checkpoint inhibitors [19,24,26,81,82]. From the data of 1556 resected gastric cancer patients conducted in an individual-patient-data meta-analysis, 121 (7.8%) had MSI-H, 576 were European patients, and 980 were Asian patients [24]. MSI-H patients had a robust favorable survival compared with those with MSI-low/microsatellite stable (MSS) disease (5-year OS, 77.5% vs. 59.3%). Conversely, patients with MSI-H did not benefit from chemotherapy plus surgery compared with surgery only (5-year OS, 75% vs. 83%; HR, 1.50). Furthermore, another meta-analysis of resected gastric cancer patients reported adjuvant chemotherapy prolonged the survival of dMMR/MSI-H patients compared with surgery alone (5-year OS, 73.5% vs. 59.7%) [19]. However, whether chemotherapy should be omitted after surgery in patients with dMMR/MSI-H is controversial. The NCCN guidelines recommend universal testing for MSI using polymerase chain reaction/next-generation sequencing methods or MMR by IHC in all newly diagnosed gastric cancer patients [25].

In this era of immunotherapy, perioperative immune checkpoint inhibitors have been evaluated in numerous prospective clinical trials. In the DANTE study, a pathologic complete response or TRG1a/b was higher in patients with MSI-H in the atezolizumab combination group compared with the FLOT group (80% [8/10] vs. 59% [7/12]) [70,83]. Moreover, the NEONIPIGA trial evaluated nivolumab plus ipilimumab as a perioperative therapy for MSI-H/dMMR locally advanced gastric cancer [84] and reported extremely favorable results, with a postoperative pathologic complete response rate of 59% (17/29 patients) and no cases of recurrence at the time of analysis.

In metastatic settings in prospective clinical trials, the MSI-H status is a predictive biomarker of immune checkpoint inhibitors. The results of the KEYNOTE-059, KEYNOTE-061, and KEYNOTE-062 trials demonstrated patients with MSI-H tumors in the pembrolizumab group had a markedly high survival rate (OS, not reached) [82]. In the phase II KEYNOTE-158 trial, pembrolizumab monotherapy was investigated in patients with pretreated non-colorectal cancers with MSI-H/dMMR, and demonstrated clinically meaningful benefit (ORR, 37.2% for 94 patients) [81]. Based on these results, pembrolizumab was approved in the USA and Japan for patients with previously-treated MSI-H/dMMR tumors, including advanced gastric cancer. The GARNET trial evaluated the anti-PD-1 antibody dostarlimab and reported an ORR of 38.7% in dMMR patients with non-endometrial solid tumors (ORR, 37.5% in AGC) [85], and eventually dostarlimab was granted accelerated approval by the FDA.

A subgroup analysis of the CheckMate 649 trial demonstrated an extremely favorable prognosis for nivolumab plus chemotherapy or ipilimumab in patients with MSI-H tumors [26]. In addition, OS benefit was observed in patients with MSI-H tumors with nivolumab plus chemotherapy versus chemotherapy (unstratified HR, 0.38), and the ORR was also higher in the nivolumab plus chemotherapy arm than in the chemotherapy arm (ORR, 55% vs. 39%). Similarly, nivolumab plus ipilimumab demonstrated a longer median OS (unstratified HR, 0.28) and higher ORR (70% vs. 57%).

In summary, it is unclear whether the use of immune checkpoint inhibitors for MSI-H gastric patients can replace surgery in a locally advanced stage or cytotoxic chemotherapy in the metastatic setting.

### 9.3. Tumor Mutational Burden

The tumor mutation burden (TMB) is a useful biomarker of the efficacy of immune checkpoint inhibitors to treat various cancers [86]. TMB quantifies the number of somatic mutations per coding region of the genome and can be measured clinically using next-generation sequencing. MSI-H gastric cancer has a high TMB (TMB-H), which promotes a robust immune cell response [17,87,88]. In the KEYNOTE-158 trial, TMB-H solid tumors had a higher ORR compared with non-TMB-H tumors (ORR, 30.3% vs. 6.7%). After MSI-H tumors were excluded from the analysis, TMB-H still had a high tumor response (ORR, 27.1%) [81,89]. In the KEYNOTE-061 trial, an exploratory analysis demonstrated that pembrolizumab had survival benefits for the TMB-H cohort compared with paclitaxel and that the clinical efficacy remained after excluding MSI-H patients [90]. Furthermore, TMB-H remained a positive predictor compared with CPS. Based on these results, pembrolizumab was granted approval in the USA and Japan for TMB-H solid tumors (≥10 mutations/Mb). However, a retrospective cohort study reported advanced gastric cancer patients with TMB-H treated with systemic therapy in clinics had better outcomes compared with those with a lower mutational burden [91,92]. Therefore, whether TMB-H is an independent or useful biomarker for gastric cancer patients requires further investigation using a large cohort.

### 9.4. EBV

EBV-positive gastric cancer is associated with high CD8-positive T cell infiltration and PD-L1/L2 expression, indicating its sensitivity to immune checkpoint inhibitors. Several retrospective cohort studies reported that PD-L1 expression on tumor cells was associated with EBV positivity [73,93,94]. In addition, the induction of interferon-γ by interferon regulatory factor 3 activation had a critical role in immune responses in EBV-positive gastric cancer patients [95]. Furthermore, a phase II study of pembrolizumab demonstrated EBV-positive gastric cancer was susceptible to treatment with immune checkpoint inhibitors [31].

### 9.5. Investigational Biomarkers

DNA polymerase epsilon (*POLE*) has a primary role in DNA proofreading and replication, and alterations in *POLE* lead to hypermutated tumors, resulting in antitumor responses to immune checkpoint inhibitors [96]. Of 14,229 patients with solid tumors, 486 (3.4%) had a *POLE*-aberrant tumor. Patients with pathogenic *POLE* mutations who were treated with immune checkpoint inhibitors showed improved survival rates (median OS, 29.5 vs. 6.8 months) compared with those with benign variants. A retrospective gastric cancer cohort study revealed the total frequency of *POLE* mutations was 7.99% [97]. Moreover, *POLE* mutations were associated with dMMR, PD-L1 overexpression, and TMB-H, suggesting they might be a specific biomarker for advanced gastric cancer.

Epigenetic alterations were reported to be potential negative predictors of immune therapy in gastric cancer. Alternative promotor alterations were associated with the reduced activity of T cells and decreased immunity, reducing the survival rate of patients with promotor alterations compared with those with no promoter alterations who were treated with immune checkpoint inhibitors (median PFS, 55 days vs. 121 days) [98,99]. 

## 10. Regimen Selection Strategies for HER2-Negative Gastric Cancer in a First-Line Setting: Immunochemotherapy Combination versus Chemotherapy Alone

In the era of immunochemotherapy and approval for HER2-negative advanced gastric cancer as first-line therapy, there is only a general consensus that a combination of chemotherapy plus anti-PD-1/PD-L1 antibodies should be used for gastric cancer patients with CPS ≥ 5. Considering the optimal regimens for HER2-negative chemotherapy-naïve advanced gastric cancer patients with CPS < 5 tumors, the addition of anti-PD-1/PD-L1 antibody to chemotherapy may depend on a patient’s general condition, personal or institutional experience, approval status, adverse events (AEs), cost-effectiveness, and accessibility to subsequent immunotherapy. Indeed, subsequent therapy was reported to be associated with a better survival rate [100,101]. However, of 10,581 advanced gastric cancer cases who received palliative systemic therapy in Japan, only 2930 (22.5%) underwent third-line chemotherapy [102,103]. If patients who did not receive immunotherapy in first-line treatment are unable to receive third-line treatment due to a poor general condition, they might not be able to receive immunotherapy during the entire course of palliative chemotherapy. One reason for not being able to receive subsequent systemic therapy is insufficient oral intake due to a primary tumor or peritoneal dissemination, thus requiring multidisciplinary care such as surgical gastrojejunostomy or endoscopic stent placement [104,105,106,107]. Data on immunochemotherapy for patients not eligible for clinical trials, such as poor performance status, insufficient oral intake, and disseminated intravascular coagulation syndrome, are lacking. When translating the results of clinical trials conducted with eligibility criteria into clinical practice, the significance of a new drug should not be determined simply by whether a statistically significant difference was observed, rather, it is important to consider multiple perspectives and have a careful consultation and discussion with each patient. Further investigations are warranted to adapt optimal treatment approaches for advanced gastric cancer with a focus on molecular biomarkers other than CPS as well as clinicopathological features.

## 11. Potential Regimens and Future Directions

### 11.1. Anti-PD-1/PD-L1 Antibodies Plus Multikinase or Vascular Endothelial Growth Factor Inhibitors

Paclitaxel plus ramucirumab is the standard of care for advanced gastric cancer patients in a second-line setting [108,109]. The simultaneous inhibition of PD-1 and vascular endothelial growth factor (VEGF) pathways enhances T cell mobilization, induces immune activity in the tumor microenvironment, and synergizes with other antitumor effects [110]. A single arm Phase II study of nivolumab with paclitaxel plus ramucirumab reported favorable antitumor activity (ORR, 37.2%; 6-month PFS rate, 46.5%) [111]. Furthermore, data from the ATTRACTION-2 trial of a Japanese intention to treat population indicated better clinical effects of nivolumab with prior ramucirumab use [112]. Most recently, avelumab with paclitaxel plus ramucirumab in the phase II RAP trial showed better outcomes compared with a Western population in the RAINBOW trial (median OS, 10.6 vs. 8.6 months) [113]. Other combinations of immune checkpoint inhibitors with VEGF-targeted therapy have been reported. Regorafenib, a potent inhibitor of angiogenic and oncogenic kinases, plus nivolumab demonstrated durable responses in previously-treated advanced gastric cancer (ORR, 44%) [114]. Lenvatinib, a multikinase inhibitor of VEGF receptors and other receptor tyrosine kinases, plus pembrolizumab showed promising clinical activity in advanced gastric cancer patients (ORR, 69%) [115]. Several other phase III trials are investigating combinations of multikinase inhibitors with immune checkpoint inhibitors in advanced gastric cancer patients (INTEGRATEIIB trial, NCT04879338; LEAP-015 trial, NCT04662710).

### 11.2. CAR-T

Currently, chimeric antigen receptor (CAR)-T-cell targeted therapy is being developed for the treatment of solid tumors [116]. Within the GS subtype, approximately 15% of cases had *CLDN18–ARHGAP* alterations [17]. A phase I study of CLDN18.2-targeted CAR-T cells in CLDN18.2-positive gastrointestinal cancer patients, including gastric cancer, showed promising clinical activity (ORR 57.1%; 6-month OS, 81.2%). Targeted CAR-T therapy, a novel immunotherapy approach different from immune checkpoint inhibitors, is expected to become a personalized treatment strategy for advanced gastric cancer in the future.

## 12. Conclusions

Adding anti-PD-1/PD-L1 antibody to chemotherapy as first-line therapy demonstrated clinical benefit for HER2-negative advanced gastric cancer. However, selecting optimal regimens for patients with CPS < 5 tumors requires the consideration of various factors such as the patient’s general condition, including their PS, age, MSI status, personal or institutional experience, approval status, AEs, cost-effectiveness, and accessibility to subsequent immunotherapy. For HER2-positive advanced gastric cancer in upfront settings, a combination of pembrolizumab with trastuzumab plus chemotherapy showed promising efficacy, and other combinations of anti-HER2 therapy and immune checkpoint inhibitors have indicated promising effects. In a prospective setting, immune checkpoint inhibitors for MSI-H/dMMR locally advanced gastric cancer had extremely favorable efficacy. A novel immunotherapy approach using CAR-T cell therapies might be used as a personalized treatment for advanced gastric cancer. Despite these breakthroughs, there is still an urgent need to establish novel biomarkers for immune therapy and develop new immunotherapies.

## Figures and Tables

**Table 1:** Comparison between the East and West gastric cancer.

	East Asia	West
Prevalence	High	Low
Proportion of gastroesophageal junction cancer	Low	High
Early cancer	Common	Rare
Helicobacter pylori-induced gastric cancer	Common	Rare
Standard surgery	D2 dissection	D1-D2 dissection
5-year survival by surgery	70%	30–40%
Standard adjuvant chemotherapy	Post-operative chemotherapy in Japan (S-1/CapeOX/SOX/DS)	Perioperative chemotherapy (Neo and post adjuvant FLOT)
Standard chemotherapy regimen for advanced gastric cancer	Doublet	Doublet or triplet

Abbreviation: CapeOX, capecitabine + oxaliplatin; SOX, S-1 + oxaliplatin; DS, docetaxel + S-1; FLOT, 5-FU + leucovorin + oxaliplatin + docetaxel.

**Table 2:** Overview of clinical trials including immune checkpoint inhibitors.

Line	Agent	Target	Patient Selection	Trial	Phase	Experimental Arm	Control Arm	Key Results	Approval (USA/EU/JPN/KOR/CHN/TWN)
Locally advanced	Atezolizumab	PD-L1	Perioperative	DANTE	II	Atezo + FLOT → op → atezo + FLOT	FLOT → op → FLOT	Awaited: High pathological response in higher PD-L1 CPS and MSI-high	
	Nivolumab	PD-1	pStage III	ATTRACTION-5	III	Adj nivo + S-1/CapeOX	Adj S-1/CapeOX	Ongoing	
	Ipilimumab+ nivolumab	CTLA-4 + PD-1	Comp preop CTx	VESTIGE	II	Adj ipi+ nivo	Adj CTx	Ongoing	
	Pembrolizumab	PD-1	Perioperative	KEYNOTE-585	III	Pembro + XP/CF/FLOT → op → pembro + XP/CF/FLOT	Placebo	Ongoing	
	Durvalumab	PD-L1	Perioperative	MATTERHORN	III	Durva + FLOT→ op → durva + FLOT	Placebo	Ongoing	
	Ipilimumab + nivolumab	CTLA-4 + PD-1	MSI-H or dMMR	NEONIPIGA	II	Ipi + nivo → op → nivo	–	High CR rate (58.6%), no patient relapse	
First-line	Nivolumab	PD-1	HER2-	CheckMate 649	III	Nivo + CapeOX/FOLFOX	CapeOX/FOLFOX	Positive: OS Δ3.3 months (CPS ≥ 5), OS Δ2.2 months (all randomized)	USA, EU (CPS ≥ 5), JPN, KOR, CHN, TWN
	Ipilimumab + nivolumab	CTLA-4 + PD-1			III	Ipi + nivo	CapeOX/FOLFOX	Negative	
	Nivolumab	PD-1	HER2-	ATTRACTION-4	III	Nivo + SOX/CapeOX	SOX/CapeOX	Positive for PFS/negative for OS	
	Pembrolizumab	PD-1	HER2-	KEYNOTE-062	III	Pembro + XP/CF	XP/CF	Negative	
					III	Pembro		Noninferior for OS (CPS ≥ 10)	USA (CPS ≥ 10)
	Pembrolizumab	PD-1	HER2-	KEYNOTE-859	III	Pembro + CapeOX/CF	Placebo	Positive: Press release only	
	Avelumab	PD-L1	HER2-	JAVELIN Gastric 100	III	Avel maintenance	CapeOX/FOLFOX cont,	Negative	
	Sintilimab	PD-1	HER2-	ORIENT-16	III	Sinti + CapeOX	Placebo	Positive: OS Δ5.5/Δ2.9 months (CPS ≥ 5/any PD-L1)	CHN
	Tislelizumab	PD-1	HER2-	RATIONALE 305	III	Tisle + CapeOX/CF	Placebo	Positive: Press release only	
	Ipilimumab + nivolumab	CTLA-4 + PD-1	HER2-	MOONLIGHT	II	Ipi + Nivo + FOLFOX/ Nivo + FLOT	FOLFOX → Ipi + nivo	Median OS 16.46 months in Ipi + Nivo + FOLFOX (CPS ≥ 1)	
	Pembrolizumab + lenvatinib	PD-1 + multikinase	HER2-	LEAP-015	III	Pembro + lenva + CapeOX/FOLFOX	CapeOX/FOLFOX	Ongoing	
	Pembrolizumab	PD-1 + HER2	HER2+	KEYNOTE-811	III	Pembro + trastuzumab + CF/CapeOX/SOX	Placebo	OS awaited; ORR, 74.4% vs. 51.9%	USA
	Retifanlimab/tebotelimab + margetuximab	PD-1 (+LAG-3) + HER2	HER2+ and CPS ≥ 1	MAHOGANY	III	Reti/tebote + marge +/− CTx	Trastuzumab + CTx	On going: ORR, 64.8% in Reti + marge arm	
	Durvalumab + T-DXd	PD-L1 + HER2	HER2+	DESTINY-Gastric03	Ib/II	T-DXd ± durva ± CTx	–	Ongoing: ORR, 42.9~50%	
	Ipilimumab + nivoluamb	CTLA-4 + PD-1 + HER2	HER2+	INTEGA	II	Ipi + nivo + trastuzumab	Nivo + trastuzumab + FOLFOX	12 mo OS rate, 57% vs. 70%	
First or second-line	Pembrolizumab + lenvatinib	PD-1 + multikinase	all	EPOC1706	II	Pembro + Lenva	–	ORR, 69%	
Second-line	Pembrolizumab	PD-1	CPS ≥ 1	KEYNOTE-061	III	Pembro	Paclitaxel	Negative	
	Durvalumab ± tremelimumab	PD-L1± CTLA-4	all	DURIGAST	II	Durva + treme + FOLFIRI	Durva + FOLFIRI	Negative: PFS at 4 mo, 57.8% vs. 44.7%	
	Nivolumab + paclitaxel + ramucirumab	PD-1 + VEGF	all	-	II	Nivo + paclitaxel + ramucirumab	–	6-month OS rate, 46.5%	
	Avelumab + paclitaxel + ramucirumab	PD-L1 + VEGF	all	RAP	II	Ave + paclitaxel + ramucirumab	–	6-month OS rate, 71.2%	
Second or later line	Nivolumab ± ipilimumab	PD-1 ± CTLA-4	all	CheckMate 032	II	Nivo ± ipi	–	ORR, 8~24%	
	Pembrolizumab	PD-1	MSI-H or dMMR	KEYNOTE-158	II	Pembro	–	ORR, 45.8% (gastric cancer)	USA, JPN
	Pembrolizumab	PD-1	TMB-H		II			ORR, 28% (not MSI-H/dMMR)	USA, JPN
	Dostarlimab	PD-1	dMMR or *POLE* mut	GARNET	II	Dostar	–	ORR, 38.7%	USA
	Nivolumab + regorafenib	PD-1 + multikinase	all	INTEGRATEIIB	III	Nivo + rego	Chemotherapy	Ongoing	
Third-line	Avelumab	PD-L1	all	JAVELIN Gastric 300	III	Ave	Irinotecan or taxane	Negative	
Third or later-line	Nivolumab	PD-1	all	ATTRACTION-2	III	Nivo	Placebo	Positive	JPN, KOR, CHN, TWN
	Pembrolizumab	PD-1	all	KEYNOTE-059	II	Pembro	–	ORR, 15.5% (CPS ≥ 1)	Withdraw Indication in USA (CPS ≥ 1)
	Nivolumab + regorafenib	PD-1 + multikinase	all	REGONIVO	II	Nivo + rego	–	ORR, 44%	
	Pembrolizumab + lenvatinib	PD-1 + multikinase	all	LEAP-005	II	Pembro + lenva	–	ORR, 10%	

Abbreviation: PD-1, programmed death-ligand 1; PD-L1, programmed cell death protein 1; CTLA-4, cytotoxic T-lymphocyte-associated protein 4; adj, adjuvant; atezo, atezolizumab; FLOT, fluoropyrimidine + leucovorin + oxaliplatin + docetaxel; comp preop, completed preoperative; ipi, ipilimumab; nivo, nivolumab; pembro, pembrolizumab; XP, capecitabine + cisplatin; CR, complete response; MSI-H, microsatellite instability high; dMMR, mismatch repair deficient; HER2, human epidermal growth factor receptor 2; OS, overall survival; OS Δ, increment in OS; CPS, combined positivity score; CTx, chemotherapy; durva, duruvalumab; treme, tremelimumab; FOLFOX, fluoropyrimidine + leucovorin + oxaliplatin; SOX, S-1 + oxaliplatin; PFS, progression-free survival; CF, cisplatin + fluoropyrimidine; avel, avelumab; sinti, sintilimab; tisle, tislelizumab; LAG-3, lymphocyte activation gene-3; T-DXd, trastuzumab deruxtecan; ORR, overall response rate; mo, months; FOLIFIRI, fluoropyrimidine + leucovorin + irinotecan; lenva, lenvatinib; TMB-H, tumor mutation burden high; dostar, dostarlimab; POLE, DNA polymerase epsilon; mut, mutation; rego, regorafenib; USA, United States of America; EU, European Union; JPN, Japan; KOR, Republic of Korea; CHN, China; TWN, Taiwan; VEGF, vascular endothelial growth factor.

## Data Availability

The data that support the findings of this study are available from the corresponding author upon reasonable request.

## Abbreviations

PD-1: programmed cell death (PD)-1 protein; PD-L1, programmed cell death ligand 1; CTLA-4, cytotoxic T-lymphocyte-associated protein 4; DNA mismatch repair deficient, dMMR; TCGA, Cancer Genome Atlas Research Network; EBV, Epstein–Barr virus; MSI-H, microsatellite instability high; CIN, chromosomal instability; GS, genomically stable; ACRG, Asian Cancer Research Group; DFS, disease-free survival; OS, overall survival; CPS, combined positive score; XP, capecitabine plus cisplatin; CF, cisplatin plus fluoropyrimidine; PFS, progression-free survival; CI, confidence interval; CapeOX, capecitabine + oxaliplatin; ORR, overall response rate; FOLFOX, fluoropyrimidine + leucovorin + oxaliplatin; SOX, S-1 + oxaliplatin; FDA, the Food and Drug Administration; Treg, regulatory T cells; ADCC, antibody-dependent cellular cytotoxicity; HER2, Human Epidermal Growth Factor Receptor 2; T-DXd, trastuzumab deruxtecan; TRAEs, treatment related adverse events; TPS, tumor proportion score; MSS, microsatellite stable; TMB, Tumor mutation burden; POLE, DNA polymerase epsilon; VEGF, vascular endothelial growth factor; CAR, chimeric antigen receptor.
